# Bacterial live therapeutics for human diseases

**DOI:** 10.1038/s44320-024-00067-0

**Published:** 2024-10-23

**Authors:** Elisabet Frutos-Grilo, Yamile Ana, Javier Gonzalez-de Miguel, Marcel Cardona-i-Collado, Irene Rodriguez-Arce, Luis Serrano

**Affiliations:** 1 Centre for Genomic Regulation (CRG), The Barcelona Institute of Science and Technology, Barcelona, Spain; 2 Universitat Pompeu Fabra (UPF), Barcelona, Spain; 3 ICREA, Pg. Lluis Companys 23, Barcelona, Spain

**Keywords:** Live Therapeutic Products, Pathogenic Bacteria, Microbiota, On-Site Delivery, Gut-Body Axis, Biotechnology & Synthetic Biology, Microbiology, Virology & Host Pathogen Interaction

## Abstract

The genomic revolution has fueled rapid progress in synthetic and systems biology, opening up new possibilities for using live biotherapeutic products (LBP) to treat, attenuate or prevent human diseases. Among LBP, bacteria-based therapies are particularly promising due to their ability to colonize diverse human tissues, modulate the immune system and secrete or deliver complex biological products. These bacterial LBP include engineered pathogenic species designed to target specific diseases, and microbiota species that promote microbial balance and immune system homeostasis, either through local administration or the gut-body axes. This review focuses on recent advancements in preclinical and clinical trials of bacteria-based LBP, highlighting both on-site and long-reaching strategies.

## Abstract (web summary)

This Review discusses recent advancements in preclinical and clinical trials of bacteria-based live biotherapeutic products, highlighting both on-site and long-reaching strategies.

## Graphical abstract

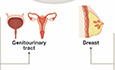

## Introduction

Advancements in synthetic and systems biology have opened new avenues for utilizing microorganisms as live biotherapeutic products (LBP) (FDA, 2016). The appeal of bacteria as LBP lies in their ability to colonize specific human tissues and adapt to the environment to continuously produce and deliver multiple therapeutic agents locally. This reduces dosage requirements, unspecific adverse effects related to systemic administration and production costs.

Bacteria offer a diverse range of therapeutic possibilities for infectious and non-communicable diseases. Both pathogenic and microbiota species have been explored. Pathogenic bacteria are engineered to target specific diseases and are usually administrated on the target tissue to secrete biologics or stimulate the immune system on-site. Microbiota-based therapeutics aim to restore microbial balance, promote overall health, and maintain immune system homeostasis. Beyond local regulation, researchers are investigating the long-reaching immune impact of gut microbiota. The term “axis” denotes the bi- or multi-directional pathways facilitating biochemical communication between various parts of the body. The mesenteric lymphatic system plays a crucial role in this communication, allowing intact bacteria, their fragments or their metabolites to cross the intestinal barrier, reach systemic circulation, and influence on distant organs, such as the breast, the liver or the brain.

In this review, we examine recent developments in bacterial LBP, distinguishing between direct on-site bacterial treatment (pathogens or microbiota-species) and indirect therapeutic effects mediated by the gastrointestinal-body axes. We provide a comprehensive overview of significant and recent preclinical and clinical trials.

## On-target therapies

**Figure 1:**
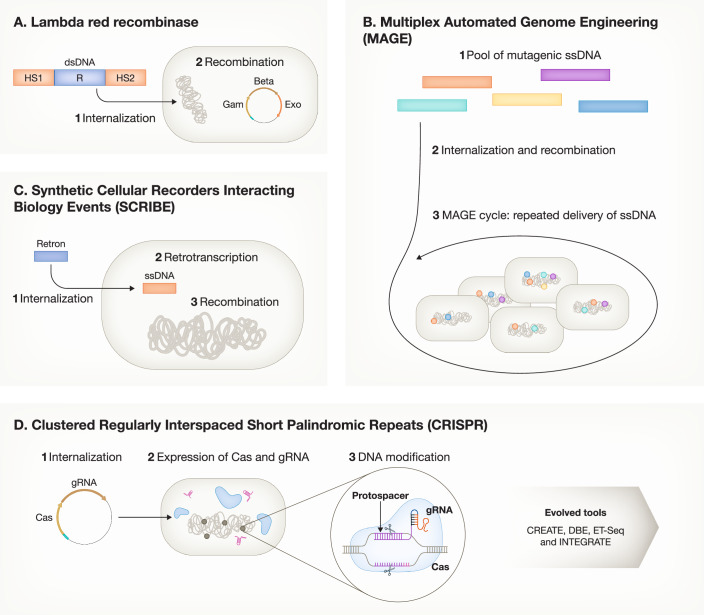
Schematic overview of the widely used genetic engineering tools for bacteria modification. (**A**) Lambda red recombinase system encompasses internalization of dsDNA recombination cassette, which contains the homology sequences (HS1 and HS2) and resistance (R), and recombination during replication. (**B**) MAGE consists of the repeated delivery of ssDNA that increases the penetrance of mutations allowing multiplex genome edition. (**C**) SCRIBE based on recombination after retron cassette internalization and retrotranscription. (**D**) CRISPR is characterized by its main components, the gRNA, protospacer-associated motif, and Cas protein. Different set up of this system can be used alone or in combination with other available methods to accomplish site-directed mutations at different scales, in multiplex fashion and with high efficiency. These techniques include: enabled trackable genome engineering (CREATE), dual base editors (DBE), enviromental transformation sequencing (ET-Seq), and insertion of transposable elements by guide RNA-assisted targeting (INTEGRATE).

Employing bacterial LBP directly on the target tissue provides a precise approach, minimizing collateral damage to the surrounding healthy tissue or systemic effects. The rapid evolution of genetic engineering tools has further enhanced this therapeutic strategy, with various technologies available, from plasmids to DNA editing platforms (Zeng et al, 2022a; Farzadfard et al, 2021) (Table 1; Fig. 1A–D). These advancements enable the adaptation of biological systems for developing LBP for untreatable or poorly prognosed medical conditions. In this respect, strategies involving both pathogenic and microbiota species have been extensively explored.

**Table 1:** Summary of tools used for bacterial genetic engineering.

Tool	Mechanism	Application/scale	Advantage	Limitations	Observations	References
**Lambda red-System**	Expression of REC/REC or Lambda red systems facilitates the recombination of customized ssDNA oligonucleotides sequences during DNA replication (recombineering). Programmable genomic integrations specified by the homology regions flanking the foreign DNA cassette.	Punctual modifications restricted to few loci.	Precise generation of insertions, deletions, and point mutations at loci specified by flanking homology regions (35 bp).	Low recombination frequency and efficiency (1 in 10^3^ –10^4^).	Used in combination with CRISPR/Cas9 to shorten edition time.	(Mosberg et al, 2010b; Zeng et al, 2022b)
**MAGE (Multiplex automated genome engineering)**	Genetic information is introduced by oligos or other carriers, allowing modifications at different chromosomal locations simultaneously. Penetration of mutations can be increased by multiple cycles of editing into edited cell populations.	Large-scale programming of different length scales (from nucleotide to genome level)	Fast, automated and high-throughput genome editing. Combinatorial genomic diversity promoted by different chromosome location in a single cell or cell population. DSBs can be avoided by adding Lambda red recombinase.	Off-target mutations. Efficiency depends on a high allelic recombination frequency in the host organism.	New generation tools based on MAGE: Cos-MAGE, TM-MAGE (fivefold reduction in off-target mutations), pORTMAGE, CRMAGE, and CMGE (combines MAGE with CRISPR).	(Gao et al, 2024; Wannier et al, 2021; Santos and Yoshikuni, 2014)
**Conjugative Assembly Genome Engineering (CAGE)**	Based on bacterial conjugation, allows horizontal DNA transfer by direct cell-to-cell contact. The machinery required is encoded on self-transmissible conjugative plasmids. It includes the proteins for recognition and capture of recipient strains (F pilus), formation of cell-to-cell contact for DNA transfer (mating apparatus) and transfer of plasmid DNA. The transferred DNA can undergoes recombination within the recipient genome.	Genome scale. Permits the construction of chimeric genomes from different strains.	Genome-wide modifications	The transfer efficiency drops with the DNA fragment size. Currently limited to *E.coli*.	CAGE can be used in combination with MAGE.	(Ma et al, 2014; Zeng et al, 2022b)
**Recombinase- Assisted Genome Engineering (RAGE)**	Recombinase-mediated cassette exchange delivered by either plasmid or phage	Integration of bacterial artificial chromosomes in different loci from the host cell. Used for the generation of large and complex biological systems.	Extended maintenance of phenotypes (over 50 generations), even in the absence of external selection (i.e., antibiotics). Enables insertions of up to 60 kb with high efficiency and a full exploration of parameters (genetic background, integration locus and copy number)	Limited to model bacteria	The derivative CRAGE can be employed in non-model bacteria.	(Santos et al, 2013; Garcia-Morales et al, 2020; Wang et al, 2019, 2020; Zeng et al, 2022a; Santos and Yoshikuni, 2014)
**Synthetic Cellular Recorders Interacting Biological Events (SCRIBE)**	Retroelement-mediated DNA writing platform for conditional and targeted editing of genomes. ssDNA is expressed intracellularly from an engineered retroelement (retron) cassette via RT and recombined into homologous sites on the genome by beta-mediated recombination.	Applicable to the entire genome. Useful as a tool for inducible genome editing and evolution of organisms. Can be used for populations editing.	Controllable bacterial genome rewriting. Not required the presence of cis-encoded elements on target	Moderate efficiency (10^−4^ edits per generation).	New generation HiSCRIBE solves SCRIBE limitations.	(Standage-Beier and Wang, 2017; Farzadfard et al, 2021; Simon et al, 2019; Farzadfard and Lu, 2014)
**Clustered regularly interspaced short palindromic repeats (CRISPR)**	DNA or gene-editing tool based on natural defense of prokaryotic organisms. Main components of CRISPR are gRNA/sgRNA and CRISPR-associated ribonuclease Cas 3/9/12. gRNA targets specific DNA sequence and guides Cas enzyme to generate DSB or SSB in DNA, generating blunt-ended, sticky-ended nick or large gaps, depending on the Cas type. Breaks can be repaired, either NHEJ or HDR, resulting in gene mutations or precise edits, respectively.	Gene or genome scale. Applied for multi-loci editing	High efficiency. Different Cas proteins and DNA repair systems can be selected to achieve efficient knockout or insertion of target genes. It can be applied to insertions, deletions or mutations.	Toxicity of Cas9/12 limits its application to all bacteria. Cas9: high off-target rate. Cas12: weak cleavage activity. Limited development of endogenous repair systems.	Simultaneous transcriptional activation (CRISPRa), repression (CRISPRi) or deletion (CRISPRd) are feasible by optimizing sgRNA constructs expression. New CRSIPR-based technologies include: CREATE strategy (MAGE + CRISPR/Cas9 and barcoding), DBE (C and A edition), ET-Seq, and programmable integrase systems, that is an optimized insertion of transposable elements by guide RNA–assisted targeting system (INTEGRATE).	(Ding et al, 2020; Lian et al, 2019; Zeng et al, 2022b; Shelake et al, 2023; Zhang et al, 2024; Rubin et al, 2021; Hillary and Ceasar, 2023; Vo et al, 2021; Strecker et al, 2019)

Double Strand Break (DSB), Chassis-independent RAGE (CRAGE), single stranded DNA (ssDNA), Retrotranscription (RT), High-efficiency SCRIBE (HiSCRIBE), Single guide RNA (sgRNA), Single Strand Break (SSB), Non-Homologous End-Joining Repair (NHEJ), Homology-Directed Repair (HDR), CRISPR EnAbled Trackable genome Engineering (CREATE), Dual Base Editors (DBE), Enviromental Transformation Sequencing (ET-Seq), Adenine (A), Cytosine (C).

Advantages and limitations are listed together with their applications.

### Bacterial pathogens

**Figure 2:**
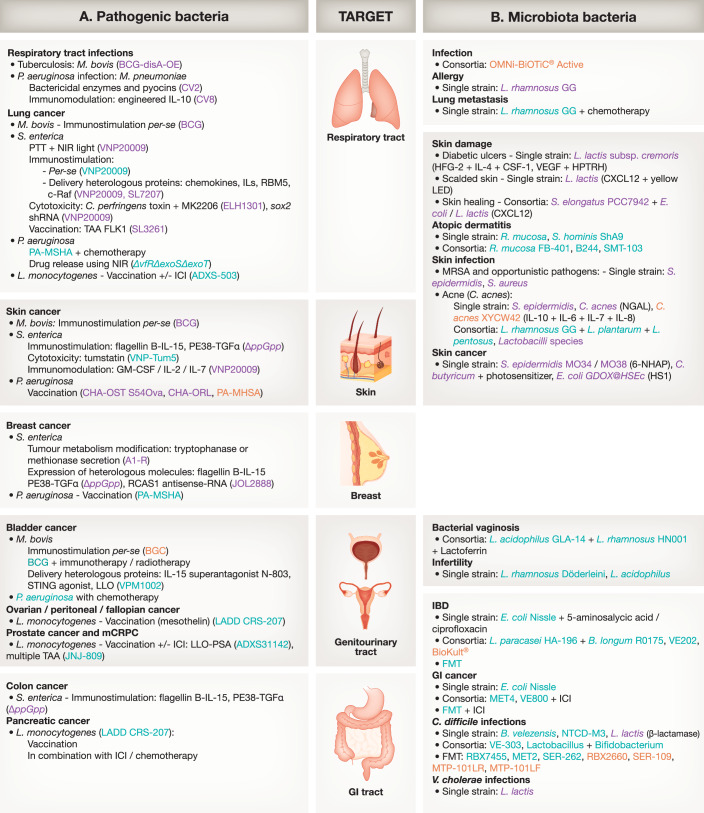
Bacteria-based LBP used for on-target treatment. LBP based on pathogenic bacteria (left), or microbiota (right) are shown. Stage of development is indicated in colors: preclinical (purple), clinical (green) and approved (orange). Immune checkpoint inhibitor (ICI), nicotinamide mononucleotide (NM), fecal microbiota transplantation (FMT), arginyl-glycyl-aspartic acid peptide motif (RGDPM), photothermal therapy (PTT), near-infrared light (NIR), mannose-sensitive-hemagglutinin (MSHA), 6-*N*-hydroxyaminopurine (6-NHAP), tumor-associated antigen (TAA), prostate-specific antigen (PSA), heparin-poloxamer thermo-responsive hydrogel (HPTRH), heparin sulfatase 1 (HS1), RNA-binding motif protein 5 (RBM5), heparin sulfatase 1 (HS1), listeriolysin-O (LLO), Phe transporter (PheP) and phenylalanine ammonia-lyase (PAL), oxalyl‐CoA synthetase gene (ScaaE3), oxalate/formate antiporter (OxlT), oxalyl‐CoA decarboxylase (OxdC), formyl‐CoA transferase (Frc) and ultrasound (US).

LBP derived from bacterial pathogens offers unique advantages, particularly in eliciting strong immune responses, potentially aiding in disease treatment. Additionally, the extensive understanding of their metabolism, pathogenic mechanisms, and virulence factors can be harnessed for therapy. We summarize key pathogenic bacteria used as LBP in preclinical studies and clinical trials (Fig. 2A; Dataset EV1).

#### *Mycobacterium bovis* (BCG)

Bacillus Calmette-Guerin (BCG) is an attenuated form of *Mycobacterium bovis* that was developed over a century ago as a vaccine for tuberculosis (Cardillo et al, 2021b). It has been proposed to work through the activation of CD4 T cells in the local tumoral environment (Antonelli et al, 2020).

##### Cancer

BCG has gained FDA approval as an LBP for non-invasive bladder cancer (deKernion et al, 1985; Lamm et al, 1980). Current clinical trials are exploring strategies to enhance BCG efficacy in this condition, including increasing induction courses and combining BCG with immunotherapy or radiotherapy (NCT02281383, NCT05580354, NCT03892642, NCT03711032, NCT04149574, NCT03317158). In the engineered BCG chassis, VPM1002 urease C is substituted by the Listeria gene listeriolysin-O (LLO). This leads to a phagosome more prone to fuse to the lysosome when BCG is engulfed by macrophages and promotes antigen release to the cytosol to enhance the immunopotency of BCG (Nieuwenhuizen et al, 2017). Patients affected with recurrent non-invasive bladder cancer after conventional BCG treatment were recurrence-free in half the cases when treated with VPM1002 (NCT02371447) (Rentsch et al, 2022). Other approaches involve engineered BCG expressing heterologous proteins such as interleukin (IL)-15 superagonist N-803, or STING agonist (NCT03022825) (Chamie et al, 2023). Additionally, BCG effects were explored in other cancer types, such as melanoma, leukemia, and lung cancer (Cardillo et al, 2021a; Benitez et al, 2019). In these studies, the intravenous administration of BCG showed stimulation in the immune system response per-se in animal models (Moreo et al, 2023). In a preclinical context, BCG expressing IL-2 has also been effective in Epstein-Barr virus-associated tumors (Yu et al, 2024).

##### Tuberculosis

BCG overexpressing *disA* (BCG-*disA*-OE) enhanced the STING pathway activation, which results in NF-κB and IRF3-dependent cytokine production, showing efficacy in preclinical tuberculosis models (Singh et al, 2022).

#### *Salmonella enterica* serovar Typhimurium

Salmonella is a diverse genus of Gram-negative bacteria, spanning various species known to cause a spectrum of diseases. However, *S. enterica* serovar Typhimurium exhibits interesting abilities for the potential treatment of cancer (Becerra-Báez et al, 2022)*. S. enterica* demonstrates natural tropism for tumors, promoting a natural and potent anti-tumor effect through mechanisms including direct competition within the tumor microenvironment (TME), production of cytotoxic molecules, induction of cellular stress and activation of immune responses via recognition of pathogen-associated molecular patterns (Grille et al, 2014; Zhang et al, 2007; Kim et al, 2015). Different attenuated strains of *S. enterica* (Δ*ppGpp*, VNP20009, ELH1301) have been designed to synthesize and/or secrete biomolecules to treat different tumors:

##### Lung cancer

*S. enterica* strains have been engineered to deliver immunomodulatory molecules such as CCL21 or IL-18 (Loeffler et al, 2009, 2008). In a similar way, *S. enterica* SL7207 has been engineered for expressing c-Raf, inducing CD8 + T cells cytotoxic activity in animal models of lung adenocarcinoma (Gentschev et al, 2005). *S. enterica* VNP20009 strain carrying small hairpin RNA (shRNA) targeting the Sox2 transcription factor and a designed polypeptide HM-3 reduced cell migration and caspase-3 mediated cell apoptosis both on in vitro and in an in vivo xenograft lung cancer-mouse model (Zhao et al, 2016). *S. enterica* ELH1301 demonstrated significant efficacy against tumor cells when engineered to secrete *Clostridium perfringens* toxin. This effect was particularly pronounced when administered alongside the allosteric AKT inhibitor MK2206 in a non-small cell lung cancer model (Deb et al, 2022). *S. enterica* strain SL3261 vaccine encoding the extracellular domain of the murine VEGFR-2 (FLK1) promoted both humoral and cellular immune responses against endothelial cells and prolonged survival in a Lewis lung carcinoma model (Zuo et al, 2010). Lastly, *S. enterica* expressing the splicing-involved RNA-binding motif protein 5 (RBM5) significantly slowed down tumor growth and metastatic proliferation in a lung adenocarcinoma mice model (Shao et al, 2013).

Another therapeutical approach involves using photothermal agents to generate localized heat upon exposure to near-infrared light Proliferation of *S. enterica* VNP20009 within lung tumors induces inflammation and tumor-specific thrombosis, resulting in a darker tumor appearance with increased near-infrared absorbance. This enhances the specificity of photothermal therapy to the tumor site while minimizing damage to normal tissue (Chen et al, 2018).

##### Breast cancer

For breast cancer treatment, amino acid deprivation within tumors by secreting catalytic enzymes (tryptophanase or methionase) has been explored (Hababag et al, 2023). *S. enterica* A1-R derived from VNP20009 has garnered attention for its auxotrophy for leucine and arginine. This characteristic restricts its growth to altered tissues, showing promising results in preclinical studies of triple-negative breast carcinoma (Hamada et al, 2021). The Δ*ppGpp* mutant strain has been designed for producing fusion proteins, such as adjuvant flagellin B with IL-15, or immunotoxin PE38 linked with TGFα in breast cancer and also in colon and melanoma xenografts (Lim et al, 2017). Finally, *S. enterica* JOL2888, an engineered strain auxotrophic for tryptophan, was capable of producing a long non-coding antisense-RNA against RCAS1, which showed a significant reduction in tumor size in a mouse model of breast cancer (Sivasankar et al, 2023).

##### Melanoma

*S. enterica* VNP20009 has been engineered to incorporate the arginyl-glycyl-aspartic acid peptide motif responsible for cell adhesion to the extracellular matrix (Park et al, 2016). In another study, a targeted expression system using *S. enterica* strain VNP20009 was developed to deliver tumstatin under the control of a hypoxia-induced promoter (VNP-Tum5). The treatment with VNP-Tum5 demonstrated significant anti-tumor and anti-angiogenic activities, resulting in suppressed tumor growth and extended survival in a mouse model of B16F10 melanoma (Bao et al, 2022). VNP20009 has been also engineered to deliver immunomodulatory molecules such as GM-CSF, IL-7, or IL-2 in vivo (Lin et al, 2024; al-Ramadi et al, 2009).

#### *Pseudomonas aeruginosa*

The Gram-negative bacterium *P. aeruginosa* is known to cause lung infections in immunocompromized individuals, particularly to those with cystic fibrosis. *P. aeruginosa* colonizes lung tissues and selectively accumulates within both necrotic and viable areas of solid tumors (Pawar et al, 2015). Additionally, naturally occurring substances like bacteriocins (e.g., exotoxin A or T), azurin, and small peptides demonstrated potent anti-tumoral effects across various types of cancer cells (Pang et al, 2022; Choi et al, 2023; Fu et al, 2023).

##### Lung cancer

*P. aeruginosa* mannose-sensitive-hemagglutinin (PA-MSHA) chemically weakened was investigated in conjunction with chemotherapy (vinorelbine and cisplatin) in clinical trials for non-small cell lung cancer (Chang et al, 2015). An attenuated strain of *P. aeruginosa* PAO1 (∆*vfR*∆*exoS*∆*exoT*) was developed through metabolic engineering and optogenetics to provide control over bacterial adhesion, colonization, and drug release using NIR light in a subcutaneous lung tumor model of A549 cell line (Fu et al, 2023).

##### Breast cancer

In a Phase II clinical trial, *P. aeruginosa* MSHA was utilized for treating metastatic breast cancer, resulting in reduced side effects and enhanced immune responses via TLR4 recognition (Lv et al, 2015; Pang et al, 2022).

##### Melanoma

*P. aeruginosa* CHA-OST S54-Ova strain was generated by deleting three virulence genes (Δ*exoS*Δ*orf1*Δ*exoT*) and genetically modified to express a fusion gene encoding the N-terminal 54 amino acids of ExoS for T3SS-mediated translocation and the C-terminus of ovalbumin (OVA) for immunogenicity. This strain was able to elevate the number of OVA-specific CD8 + T cells in vivo, and the mice inoculated with CHA-OST S54-Ova were resistant to the challenge of OVA-expressing mouse melanoma cell line B16 (Epaulard et al, 2006). In a separate study, the authors developed the strain CHA-ORL (Δ*exoS*Δ*orf1*Δ*exoT*Δ*rhlIΔlasI)*, which displayed reduced toxicity and enhanced efficiency for delivering tumor-associated antigens (Epaulard et al, 2008). *P. aeruginosa* MSHA chemically weakened has been approved by the State Food and Drug Administration (People’s Republic of China) in 1998 as adjuvant therapy for melanoma and infectious diseases (Zheng et al, 2023).

##### Glioma

*P. aeruginosa* CHA-OST delivering the tumor-associated antigen Tyrosinase-related protein 2 effectively enhanced CD8 + T cell activation in a subcutaneous glioma model (Derouazi et al, 2010).

#### *Yersinia enterocolitica*

The Gram-negative bacillus *Y. enterocolitica* is responsible for infection characterized by gastrointestinal (GI) symptoms. Like other bacterial species, it possesses a natural system to deliver its cargo proteins into target cells via a type III secretion system, which can be harnessed for therapeutic delivery (Ittig et al, 2015). The attenuated strain of *Y. enterocolitica* T3P-Y058-739 is being tested in a clinical trial for the treatment of advanced solid tumors as monotherapy, or in combination with the immune checkpoint inhibitor Pembrolizumab. All patients are receiving deferoxamine as an iron source to promote bacterial survival and growth (NCT05120596).

#### *Listeria monocytogenes*

*L. monocytogenes* has been postulated as a chassis for the treatment of tumors in combination with immunotherapy due to its unique life cycle. *L. monocytogenes* can enter the cytoplasm of both phagocytic and non-phagocytic cells. In the former ones, *L. monocytogenes* escapes degradation and killing in the phagolysosome by expressing the pore-forming LLO. In non-phagocytic cells, it expresses surface proteins Internalin A/B surface proteins are able to interact with cellular receptors that guide internalization (Ding et al, 2023). Then, it is able to escape the endolysosomal compartment via the secretion of phospholipases and LLO. Both routes allow for translocation into the host’s cellular cytoplasm. The most widely used attenuated chassis is LADD (live-attenuated double-deleted) (Brockstedt et al, 2004). LADD loses the ability to enter in non-phagocytic cells and prevents cell-to-cell spread of the infection. The results showed a thousandfold decrease in toxicity in in vivo models while retaining the ability to stimulate the immune system. Interestingly, this attenuated chassis also promoted tumor-associated macrophage re-polarization toward an anti-tumoral phenotype (Lizotte et al, 2014).

##### Lung cancer

The off-the-shelf chassis ADXS-503, which provides immunity against 22 tumor-associated antigens related to non-small cell lung cancer, was tested successfully in combination with Pembromizulab in a clinical trial (NCT03847519).

##### Genitourinary cancers

Tumor-associated antigens exogenously expressed by *L. monocytogenes* are presented by the major histocompatibility complex to stimulate T cells (Ding et al, 2023). LADD CRS-207 expressing the human surface glycoprotein mesothelin has been tested in a clinical trial of ovarian, peritoneal, and fallopian cancer trial (NCT02575807) with unsuccessful results. The attenuated chassis ADXS31142, expressing a fusion protein of LLO and a human prostate-specific antigen, was tested in combination with Pembromizulab, showing good tolerance and increasing overall survival in metastatic castration-resistant prostate cancer patients (NCT02325557) (Stein et al, 2022). In the same condition, the chassis JNJ-809, which instead targeted four antigens, showed safety in clinical trials involving patients with prostate adenocarcinoma with metastasis (NCT02625857) (Drake et al, 2022).

##### Gastrointestinal (GI) cancer

LADD CRS-207 is currently being tested in combination with immunotherapy (NCT03190265, NCT03006302), or chemotherapy (NCT01417000 andNCT02004262) to treat pancreatic cancer (Ding et al, 2023).

#### *Mycoplasma pneumoniae*

*M. pneumoniae* is a common cause of atypical pneumonia and mild extra-pulmonary pathologies in humans that has been extensively studied (Güell et al, 2009; Trussart et al, 2017; Yus et al, 2009). Recent advancements in genetic engineering have expanded the toolkit for modifying its genome, making it an attractive candidate for bacteria-based therapies (Broto et al, 2022). This genome-reduced bacterium lacks a cell wall, reducing inflammation and enabling direct secretion of biomolecules. Attenuated strains, such as CV2 (∆*mpn133*∆*mpn372*) and CV8 (∆*mpn133*∆*mpn372*∆*mpn051::gpsA* gene from *Mycoplasma penetrans*) have shown promise in preclinical models.

##### Biofilm infections

CV2 expressing dispersin B and glycylglycine endopeptidase lysostaphin has demonstrated efficacy in dissolving *Staphylococcus aureus* biofilms formed on catheters in vivo (Garrido et al, 2021). For lung treatment, CV2 was engineered to treat *P. aeruginosa* infection by delivering glycoside hydrolases and alginate lyase A1-II to degrade biofilm, along with expressing bacteriocin pyocin L1 or S5 to kill *P. aeruginosa* in mice and ex vivo in endotracheal tubes from patients with ventilator-associated pneumonia (Mazzolini et al, 2023).

##### Inflammation

The CV8 chassis successfully expressed and secreted an engineered IL-10, reducing *P. aeruginosa*-induced inflammation in an acute model of lung infection (Montero‐Blay et al, 2023).

### Microbiota species as LBP

The human microbiota is a complex ecosystem with an estimated 10–100 trillion symbiotic microorganisms (Turnbaugh et al, 2007). The largest of these communities resides in the GI tract, while other communities exist on the skin (Byrd et al, 2018), lungs (Li et al, 2024b), oral cavity (Lamont et al, 2018), and vaginal tract (Chen et al, 2021). These microbial communities exert a crucial influence on human health, with its dysbiosis linked to numerous conditions. This has spurred interest in microbiota replacement and engineering methods, which seek to modify the human host-microbiota system for therapeutic purposes. The most relevant preclinical and clinical trials are summarized (Fig. 2B; Dataset EV2).

#### Skin

Beneficial microorganisms on the skin act as a physical barrier against external invaders. The skin microbial community is primarily influenced by the physiology of the tissue site. Fluctuations in the relative abundance of bacterial taxa are linked to different skin microenvironments. Species belonging to genera such as Propionibacterium, Staphylococcus, Corynebacterium, Streptococcus and Micrococcus are among the most prevalent skin microbiota (Byrd et al, 2018).

##### Skin damage

Diabetic ulcers arise as a severe complication due to a combination of factors, including impaired circulation, heightened vulnerability to infection, and nerve damage resulting from elevated blood sugar levels. Enhancing wound healing in diabetic patients represents a critical medical necessity. Preclinical efforts have focused on engineered *Lactococcus lactis* subsp. *cremoris* strains to promote scarring. For instance, *L. lactis* secreting FGF-2, IL-4, and CSF-1 (Kurkipuro et al, 2022), as well as VEGF and a heparin-poloxamer thermo-responsive hydrogel showed an increase in fibroblast proliferation, migration and angiogenesis (Lu et al, 2021). *L. lactis* producing CXCL12, combined with yellow LED light to promote collagen synthesis accelerated wound healing, tissue restructuring, re-epithelialization, and hair follicle regeneration. This therapy reduced inflammation (IL-1β and TNF-α) and enhanced collagen formation. It also decreased skin infections caused by Ralstonia and Acinetobacter (Zhao et al, 2021). Additionally, a photoautotrophic “living material,” comprising an engineered microbial consortium consisting of *Synechococcus elongatus* PCC7942 for sucrose production via photosynthesis and a heterotrophic engineered strain of *Escherichia coli* or *L. lactis* expressing CXCL12 facilitated healing in a full-thickness rat skin defect model (Li et al, 2023).

##### Atopic dermatitis (AD)

AD stands out as the most common chronic disease, presented as an itchy and recurrent inflammatory skin condition. Common complications of AD stem from secondary infections, mainly involving *S. aureus* and Herpes Simplex Virus. Methods for treating or preventing AD through the topical application of *Roseomonas mucosa* derived from healthy patients have been developed. Initial results from a clinical trial suggest that this treatment influences glycerophospholipid synthesis, activating tissue-repair pathways and reducing subjective pruritus without adverse events (NCT03018275) (Myles et al, 2020). In another clinical trial, the efficacy of *R. mucosa* therapy was demonstrated through improvements in disease severity, epithelial enhancement, reduced *S. aureus* burden, and decreased requirement for anti-inflammatory drugs (Myles et al, 2018). Also, the commensal *Staphylococcus hominis* A9 chassis (ShA9) was employed for the inhibition of the *S. aureus* growth in AD patients (NCT05177328) (Pinto et al, 2022). Currently, a consortium comprising three therapeutic strains of *R. mucosa* (FB-401) is undergoing evaluation for AD treatment in clinical trials (NCT04936113 andNCT04504279). Furthermore, promising results came out from the Phase 2b clinical trial using B244 bacteria to treat adults with mild-to-moderate AD and moderate-to-severe pruritus. This topically-applied therapeutic ammonia-oxidizing bacteria was well tolerated and demonstrated significant efficacy (NCT04490109) (Silverberg et al, 2023). Finally, skin microbiota transplant SMT-103 showed promise in modulating the immune system of AD patients, with ongoing clinical trials assessing its safety and efficacy (NCT05003804).

##### Skin infection

Skin damage resulting from disruption of skin integrity or inflammatory disorders (i.e. AD) can lead to significant microbiota dysbiosis, promoting infection by multi-drug resistant bacteria (i.e., methicillin-resistant *S. aureus*, MRSA) or the proliferation of opportunistic pathogens (i.e., *Cutibacterium acnes*). Preclinical studies utilizing non-modified *Streptococcus epidermidis* on ex vivo skin explants have demonstrated the potential protective role of this strain against *S. aureus* infection by promoting T cell activation and the production of antimicrobial molecules (Pastar et al, 2020). In contrast with MRSA strains, *S. aureus* can use glycerol as a carbon source to produce short-chain fatty acids (SCFA). SCFA are uptaken by MRSA, thus reducing cytoplasmic pH and promoting bacterial killing, immune activation, and antibody production against MRSA (Yang et al, 2018).

*C. acnes* is the primary bacterium implicated in acne. In a clinical trial, a bacterial consortium combining skin microbiota species (*Lactobacillus rhamnosus* GG, *Lactiplantibacillus plantarum*, and *Lactiplantibacillus pentosus*) was used for its hydrating and immunomodulatory effects (NCT04216160). Preclinical studies showed that a Lactobacilli consortium reduced *C. acnes* presence and inflammation (Lebeer et al, 2022). *S. epidermidis*-based LBP decreased *C. acnes* through immune modulation by producing butyric acid or fermenting carbon sources (Huang et al, 2022). *S. epidermidis* also mitigated UV-induced inflammation, reducing photoaging (Balasubramaniam et al, 2020; Keshari et al, 2019), promoting collagen synthesis and restoring the skin elasticity (Negari et al, 2021). Other researchers have opted to leverage the inherent capabilities of *C. acnes* to colonize pilosebaceous units by modifying it as an LBP. Engineered *C. acnes* secreting NGAL reduced sebum production and *C. acnes* infection (Knödlseder et al, 2024). Additionally, *C. acnes* subsp*. defendens* XYCW42 expressing IL-6, IL-7, IL-8 and IL-10 under an inducible promoter allowed precise regulation of bacterial therapy (Rhee et al, 2023).

##### Skin cancer

In a preclinical melanoma model, *S. epidermis* strains MO34 and MO38 were able to synthesize 6-*N*-hydroxyaminopurine, inhibiting tumor growth (Nakatsuji et al, 2018). Metabolic engineering of anaerobic oncolytic bacteria *Clostridium butyricum*, labeled with a photosensitizer, demonstrated effective tumor ablation. Once injected into the tumor, *C. butyricum* proliferates in anaerobic regions, stimulating the TME. The photosensitizer on the bacteria exerts a photodynamic effect in oxygen-rich regions, aiding in melanoma removal under light irradiation (Shi et al, 2022). Another approach involves *E. coli* (GDOX@HSEc) expressing heparin sulfatase 1, which attaches doxorubicin-loaded glycogen nanoparticles to target and colonize tumors, inhibiting angiogenesis, metastasis and reducing tumor size (Yang et al, 2023a).

#### Lung

Although the lung was originally thought to be a sterile environment, mounting evidence pointed to a significant impact of microbes in both the upper and lower respiratory tracts. The most common species in the adult human lung are Firmicutes, Bacteriodetes, and Proteobacteria. The absence of these populations, particularly Lactobacillus, was correlated with disease and has therefore guided the therapeutic efforts (Yuksel et al, 2023).

##### Infection

A novel LBP combining *Lacticaseibacillus casei* AMBR2, *L. rhamnosus* GG, and *L. plantarum* WCFS has recently shown effectiveness in reducing the cytopathogenic effects of various respiratory pathogens, including Respiratory Syncytial Virus, influenza A/H1N1 and B viruses, and HCoV-229E coronavirus in vitro. *L. casei* AMBR2 was chosen for its barrier-enhancing and anti-pathogenic properties, while *L. rhamnosus* GG and *L. plantarum* WCFS1 were selected for their immunomodulatory effects. In healthy volunteers, throat spray administration of this LBP demonstrated that the lactobacilli could temporarily colonize the throat in a metabolically active state (Spacova et al, 2023, NCT04793997). A mix of 11 strains of mostly Lactobacillus and Bifidobacteria (OMNi-BiOTiC® Active) has been shown to decrease the acute phase of upper tract infections in older adults (NCT05879393) (Strauss et al, 2023).

##### Allergy

Supplementation of *L. rhamnosus* GG strain was tested in a preclinical study for allergy treatment and showed a reduction of bronchoalveolar lavage eosinophil counts, lung IL-13 and IL-5 and airway hyperreactivity (Spacova et al, 2020).

##### Cancer

A drug delivery system using a probiotic as a carrier was developed for tumor-targeted photodynamic and sonodynamic synergistic therapy. In this system, chlorin e6 nanoparticles were prepared and incorporated into *B. bifidum*, followed by the conjugation of anti-death receptor 5 antibodies (Ce6–*B. bifidum*–anti-DR5 Ab). Ce6–*B. bifidum*–anti-DR5 Ab under 671 nm laser or ultrasound irradiation generated reactive oxygen species, targeted hypoxic regions, and inhibited laryngeal tumor growth when administered intravenously (Li et al, 2022c). *L. rhamnosus* GG was also used successfully in combination with chemotherapy in a B16 model of lung metastasis and has advanced to clinical testing (Du et al, 2022; Le Noci et al, 2018).

#### Reproductive tract

The vaginal microbiota is typically low in diversity and dominated by Lactobacillus species like *Lactobacillus crispatus* (Buchta, 2018).

##### Bacterial vaginosis

It is characterized by vaginal discharge and itching, is induced by an imbalance of normal bacteria in the vagina (Mei and Li, 2022). The combination of *L. acidophilus* GLA-14 and *L. rhamnosus* HN001 plus lactoferrin improved vaginal infection symptoms (Russo et al, 2019).

##### Infertility

Genital microbiota has been evaluated as LBP to address female infertility. These LBP reduce pathogens via organic acid and hydrogen peroxide production, pH regulation, and epithelial adherence, promoting embryo implantation (Zhang et al, 2021). *L. rhamnosus* Döderleini, a commercial vaginal probiotic, combined with sperm, was used for women with recurrent miscarriages (Rafiee et al, 2022). Administration of *Lactobacilli acidophilus* before embryo transfer during the in vitro fertilization cycle significantly reduced miscarriage rates despite similar pregnancy rates (TCTR20190429001) (Thanaboonyawat et al, 2023).

#### GI tract

The gut microbiota plays a pivotal role in the host’s digestion and metabolism, as well as in the development and function of the immune system. In adults, approximately 90% of the gut bacterial microbiota belongs to the phyla Bacteroidetes and Firmicutes. The remaining 10% consists of Proteobacteria, Actinobacteria, Fusobacteria, and Verrucomicrobia (Rinninella et al, 2019).

##### Inflammatory bowel disease (IBD)

The most significant types of IBD are Crohn’s disease (Ha and Khalil, 2015) and ulcerative colitis (Segal et al, 2021). IBD patients show reduced gut microbiota diversity, losing species like Lactobacillus, Clostridium, Bifidobacterium, Faecalbacteria, and non-pathogenic *E. coli*. Most microbiota-based therapies aim to restore them by using two different approaches.

LBP as treatment. A mixture of *Lactobacillus paracasei* HA-196 and *Bifidobacterium longum* R0175 is being evaluated for improving recurrent abdominal pain (NCT02213172). VE202, a consortium of 16 Clostridia strains, promotes colonic T regulatory (T_reg_) cells and produces immunoregulatory metabolites in colitis mouse models. VE202 is currently in clinical trials for safety and colonization, providing data for planned Phase 2 trials in ulcerative colitis patients (Silber et al, 2022) (NCT05370885). Bio-Kult^®^, a mix of 14 bacterial strains, was tested in patients with diarrhea-predominant IBD, significantly reducing abdominal pain, symptoms, and bowel motions per day without serious adverse events (NCT03251625) (Ishaque et al, 2018). The infusion of fecal material from a healthy donor into the GI tract of a recipient to restore healthy intestinal flora, known as Fecal Microbiota Transplantation (FMT), is also being evaluated in preclinical and active clinical trials (NCT01560819, NCT02049502) (El-Salhy et al, 2020, Fine and Kelly, 2017). Initial results of ulcerative colitis patients who received FMT demonstrated a clinical remission rate of 32% due to the maintenance of the epithelial barrier (Villemin et al, 2024).

LBP in combination with IBD therapy. This involves the use of specific bacteria in combination with commonly used drugs to treat severe IBD but induces damage to the stomach and duodenal mucosa as adverse effects, such as *E. coli* Nissle with 5-aminosalycic acid (Park et al, 2022) or ciprofloxacin (NCT01772615). Furthermore, supplementation with Faecalbacteria is currently in a clinical trial aimed at maintaining the therapeutic effects of corticoid treatments against Crohnʼs Disease (NCT05542355).

##### Cancer

*E. coli Nissle* has been explored in several preclinical studies of cancer (Harimoto et al, 2022; Gurbatri et al, 2020; Chowdhury et al, 2019). The amenability of this bacterium to genetic engineering has resulted in innovative approaches, such as using them to label tumors with synthetic antigens for latter recognition by CAR-T cells (Vincent et al, 2023). In human studies, the ability of these bacteria to colonize colorectal tumors by oral administration has been validated (Gurbatri et al, 2024) (ACTRN12619000210178). In GI cancer research, combining bacteria with immune checkpoint inhibitors is being explored to enhance treatment response. MET4, a microbial consortium of 30 species, was safe and well-tolerated, increasing taxa like Enterococcus and Bifidobacterium and reducing primary bile acids (NCT03686202) (Spreafico et al, 2023). VE800 combined with Nivolumab was evaluated in gastric/gastroesophageal junction adenocarcinoma or microsatellite-stable colorectal cancer patients with no adverse effects (NCT04208958). Additionally, FMT plus Nivolumab was investigated in anti-PD-1-resistant GI cancers, showing good tolerance and increased microbial diversity among responders. Responders also exhibited increased IFN-γ + proliferating peripheral blood mononuclear cells and a correlation between specific bacterial species, tumor markers, and T-cell frequencies (NCT04130763).

##### Gut infections

*Clostridium difficile infections (CDI)*. Antibiotics are the primary treatment for CDI. However, about a third of patients experience recurrent CDI. Different strategies have been used. Single-strain therapies in clinical trials include *Bacillus velezensis*, which targets *C. difficile* without harming commensal bacteria (NCT04891965) (O’Donnell et al, 2022). Additionally, using spores of the non-toxigenic strain of *C. difficile* NTCD-M3 to counter vancomycin-induced dysbiosis showed success in preclinical models following fidaxomicin treatment and is now undergoing clinical trials (NCT05693077) (Gerding et al, 2015). In other study, *L. lactis* degrades β-lactams which disrupt commensal bacteria in the gut through the secretion and extracellular assembly of a heterodimeric β-lactamase. In a mouse model of parenteral ampicillin treatment, oral supplementation with this LBP minimized gut dysbiosis without affecting the ampicillin concentration in serum, precluded the enrichment of antimicrobial resistance genes in the gut microbiome, and prevented the loss of colonization resistance against *C. difficile* (Cubillos-Ruiz et al, 2022).

Rationally designed bacterial consortia VE-303, containing eight Clostridia strains, prevents gut recolonization by pathogenic *C. difficile* spores and shows better efficacy than a placebo in clinical trials (Dsouza et al, 2022, NCT04236778). Synthetic consortia of Lactobacillus and Bifidobacterium are also in development as potential therapies (NCT01680874) (Barker et al, 2017).

FMT-based therapies have been extensively explored. FDA-approved RBX2660 (Rebiotix®) is derived from healthy donor samples and contains up to 10 million organisms/mL, including Firmicutes and Bacteroidetes. It demonstrated safety and efficacy, in 80% of patients after 8 weeks, and 90% of this group remained infection-free at 6, 12, and 24 months time points (Orenstein, 2023; Dubberke et al, 2023; Khanna et al, 2022a). RBX7455, based on RBX2660, also showed effective gut colonization and CDI prevention (NCT02981316). Similarly, MET2, composed of microbiota from healthy donors, showed good tolerability and safety in clinical trials (NCT02865616) (Kao et al, 2021). Another success story in treating recurrent CDI is SER-109, composed of live purified eubacterial spores from healthy donors, being the second microbial therapy approved for this condition. In a clinical trial, recurrence of CDI was 12% in the SER-109 group compared to 40% in the placebo group after 8 weeks of treatment. Similarly, SER-262, based on spores from 12 Firmicutes strains, also reduced CDI recurrence (NCT03183128) (Khanna et al, 2022b; Mullard, 2023; Feuerstadt et al, 2022; Ford et al, 2019). Finally, FDA-approved FMT therapies include MTP-101LR and MTP-101LF for recurrent and fulminant CDI, respectively (Monday et al, 2024).

*Vibrio cholerae infections*. Cholera is a disease characterized by acute, watery diarrhea caused by the bacterium *V. cholerae*. Preclinical studies propose the oral administration of *L. lactis* to colonize the small intestine, and thereby reduce subsequent colonization by the pathogenic *V. cholerae*. In this respect, *L. lactis* reduced intestinal *V. cholerae* burden and improved survival in infected infant mice through the production of lactic acid (Mao et al, 2018).

## Bacterial LBP therapies involving the Gut-body axis

**Figure 3:**
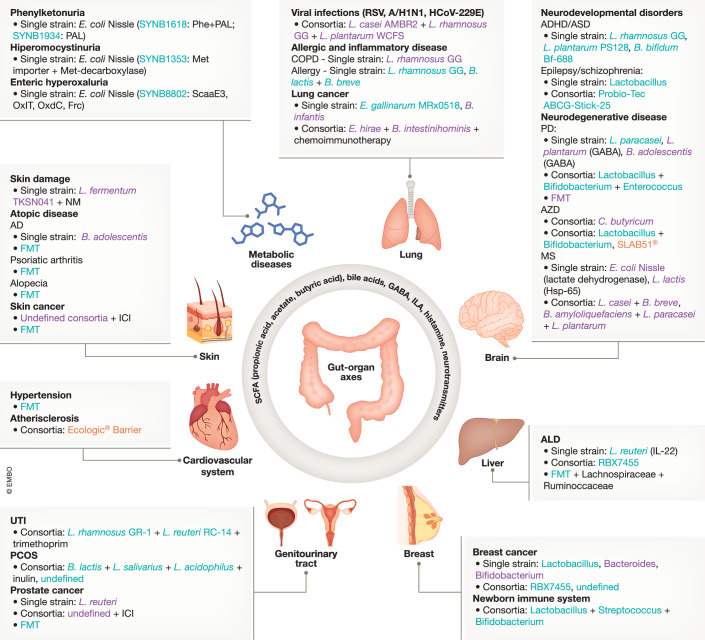
Gut-body axes: an indirect therapeutic approach for LBP administration. Stage of development is indicated in colors: preclinical (purple), clinical (green) and approved (orange). Bacterial metabolites related to the therapeutic effect are described. Immune checkpoint inhibitor (ICI), nicotinamide mononucleotide (NM), fecal microbiota transplantation (FMT), respiratory syncytial virus (RSV), chronic obstructive pulmonary disease (COPD), atopic dermatitis (AD), attention deficit hyperactivity disorder (ADHD) or autism spectrum disorder (ASD), Alzheimer’s disease (AZD), multiple sclerosis (MS), Parkinson’s disease (PD), and polycystic ovary syndrome (PCOS).

The GI is highly surrounded by lymphatic vessels and lymphoid organs forming the gut-associated lymphoid tissue (GALT). GALT allows the interconnection between the gut and the whole body, known as gut-body axes, through the translocation of gut bacteria and their metabolites (Mörbe et al, 2021; Mehrara et al, 2023). The therapeutic potential of modifying or delivering bacterial LBP in the gut to act through these specific axes has been extensively studied in preclinical and clinical trials (Fig. 3; Dataset EV3).

### Gut-lung axis

Different studies have shown that there is a conexion between the microbiome of the gut and the immune status of the lung (Li et al, 2024b).

#### Allergic and inflammatory disease

In allergic and inflammatory conditions, oral administration of *L. rhamnosus* GG showed resistance to inflammatory responses linked to cigarette smoke in Chronic Obstructive Pulmonary Disease epithelial cells (Carvalho et al, 2020). Experimental evidence, including preclinical and clinical trials, indicated that probiotic bacteria *L. rhamnosus* GG (NCT00159523), *Bifidobacterium lactis* (NCT04122495), and *B. breve* could suppress allergic and autoimmune responses, alleviate symptoms, and inhibit allergic airway reactions.

#### Lung cancer

MRx0518, an *Enterococcus gallinarum* strain, activates the immune system in responder patients receiving anti-PD-1 immunotherapy (NCT03637803) (Li et al, 2022b). Preclinical studies suggested improved outcomes in advanced lung cancer-mouse models treated with a combination of *Enterococcus hirae* and *Barnesiella intestinihominis* alongside chemoimmunotherapy (Daillère et al, 2016).

### Gut-brain axis

The gut-brain axis plays a vital role in cognitive functions and nervous system development. Recent research has highlighted the gut microbiota’s production of neuroactive molecules like γ-aminobutyric acid (GABA), serotonine, dopamine, norepinephrine, acetylcholine, and histamine, influencing the gut-brain axis network through modulation of the intestinal barrier (Cryan et al, 2019).

#### Neurodevelopmental disorders

The etiology of neurodevelopmental diseases like attention deficit hyperactivity disorder (ADHD) or autism spectrum disorder (ASD) involves genetic and early environmental factors (Wang et al, 2023). Children’s diet supplementation with *L. rhamnosus* GG or *L. plantarum* PS128 during early infancy, could notably reduce the risk of ADHD and ASD and improve the health-related quality of life of these patients (UKC-MB-KME-19-06/16) (Kumperscak et al, 2020; Liu et al, 2019). The potential impact of *Bifidobacterium bifidum* (Bf-688) supplementation on patients with ADHD has been evaluated in clinical trials (NCT04958460). Results indicated that patients receiving Bf-688 showed significant improvements in omission errors and hit reaction times in behavioral tests, unlike the placebo group. Additionally, these patients experienced a notable increase in the gut Firmicutes to Bacteroidetes ratio, which ultimately led to reduced N-Glycan biosynthesis (Wang et al, 2022). A current clinical trial is testing GABA-producing Lactobacillus safety and efficacy in epileptic seizures (NCT05539287), while others focus on probiotic cocktails or specific bacterial consortia for schizophrenia or epilepsy (Probio-Tec ABCG-Stick-25) containing four strains of probiotic microorganisms (Bifidobacteria BB-12, *L. casei*, *L. acidophilus*, and *L. rhamnosus* GG) (NCT04226898, NCT05160350).

#### Neurodegenerative diseases

Alzheimer’s disease (AZD), Parkinson’s disease (PD), and multiple sclerosis (MS) have been linked to aberrant immune activation (Mason and McGavern, 2022).

##### PD

Gut dysbiosis and accumulation of α-synuclein in the gut tract are observed in PD patients, affecting SCFA production and gastric neurons damage (Zhang et al, 2023). A clinical trial using *L. paracasei* concluded that administration of this strain could ameliorate GI symptoms on patients with PD but did not induce major changes in the global microbiota (ChiCTR1800016795) (Yang et al, 2023b). Preclinical studies suggested probiotics like *L. plantarum* or *Bifidobacterium adolescentis* could alleviate inflammation and tremors in PD (Zhong et al, 2023), by potentially producing GABA. Clinical trials are underway to assess the probiotics combination (Bifidobacterium, Lactobacillus, and Enterococcus) effect on motor symptoms in PD (NCT04871464). Recently, FMT has been used in preclinical mouse models of PD (Sun et al, 2018).

##### AZD

Research indicates a GI microbiota shift with elevated levels of *B. fragilis*, *Faecalibacterium prausnitzii*, *Eubacterium rectale*, *E. hallii*, and *Bacteroides fragilis*. This shift contributes to GI permeability and neuroinflammation, driven by bacterial derivatives (LPS and bacterial amyloids) (Varesi et al, 2022; Hauss-Wegrzyniak et al, 2000). Several preclinical studies highlighted *C. butyricum* neuroprotective potential through SCFA production (Sun et al, 2020). Clinical trials are exploring probiotic cocktails containing strains of Lactobacillus and Bifidobacterium in reducing neuroinflammation in AZD (NCT06181513). Hypoxia conditioning is being investigated as a strategy for central nervous system diseases due to its impact on amyloid precursor protein metabolism, reduction of enzyme expression that breaks down amyloid-β, and its contribution to calcium-homeostasis dysregulation in neurons (Lall et al, 2019). SLAB51® probiotic formulation showed promise in reducing nitric oxide synthesis, potentially benefiting brain oxygen supply (Bonfili et al, 2021).

##### MS

Engineered *E. coli* Nissle overexpressing lactate dehydrogenase, and *L. lactis*, expressing Hsp-65 showed immunosuppressive effects in preclinical studies on experimental autoimmune encephalomyelitis mouse models (Sanmarco et al, 2023; Guimaraes et al, 2023). The synergistic effect of *L. casei* and *B. breve* was explored in a rat model of cuprizone-induced demyelination in which *B. breve* supplementation proved more effective in reducing demyelination and oxidative stress compared to *L. casei* alone or combined (Hasaniani et al, 2024). Additional preclinical investigations evaluated the immunomodulatory potential of non-engineered probiotics like *Bacillus amyloliquefaciens*, *L. paracasei*, and *L. plantarum* on autoimmune encephalomyelitis mouse models and T cells from MS patients, showing reductions in inflammatory mediators (Ibrahim et al, 2023; Chakamian et al, 2023). FMT from MS patients in mice reproduced characteristic demyelination, thus confirming the immunoregulatory role of microbiota (Altieri et al, 2023).

### Gut-liver axis

The gut and liver are linked through the portal vein, forming the gut-liver axis crucial for molecule exchange. This axis is implicated in liver disorders like non-alcoholic fatty liver disease, alcoholic liver disease (ALD), cirrhosis, and hepatocellular carcinoma. In ALD, alcohol-related dysbiosis reduced IL-22 expression in the gut, leading to diminished REG3G protein. Engineered IL-22-expressing *L. reuteri* showed promise in reducing steatohepatitis by preventing bacterial translocation to the liver (Hendrikx et al, 2019). ALD therapies also include FMT with Lachnospiraceae and Ruminococcaceae (NCT03152188).

### Gut-breast axis

The gut-breast conexion plays a significant role in breast health and disease development (Rodríguez et al, 2021; Ruo et al, 2021).

#### Breast cancer

Microorganisms such as Clostridia and Fusobacterium in the GI tract, contribute to breast cancer development by producing toxins, estrogen-like molecules, and inflammatory substances, leading to tissue damage and malignant transformation (Viswanathan et al, 2023; Parida et al, 2021; Parida and Sharma, 2019; Urbaniak et al, 2016; Hieken et al, 2016). Preclinical and clinical trials explored the role of Lactobacillus in restoring breast microbiota and its impact on cancer prognosis (NCT03290651). *Bifidobacterium infantis 35624* in milk was orally administered to a xenograft model of breast cancer in mice, either alone or in combination with the chemotherapy drug doxorubicin (DOX). The combination of *B. infantis* milk and DOX demonstrated the strongest anti-tumor effect, significantly reducing tumor volumes (Akbaba et al, 2023). Another study introduced a novel approach that combines albumin-encapsulated DOX nanoparticles coated with chitosan, alongside anaerobic *B. infantis* as self-propelled carriers (Bif@BDC-NPs) for breast cancer chemotherapy. Upon oral administration, Bif@BDC-NPs precisely targeted the hypoxic regions within tumor tissue, enhancing drug accumulation at the tumor site. This strategy led to a remarkable tumor inhibition, significantly prolonged the median survival of the mice, and reduced the toxic side effects typically associated with DOX (Li et al, 2024a). Other trials have investigated immune system stimulation through a gut probiotic cocktail before breast surgery, using again RBX7455 (NCT04139993) or a consortium of Lactobacillus, Bifidobacterium, and yeast (NCT03358511) (Nandi et al, 2023).

#### Newborn immune system

The gut-breast axis also plays a crucial role during breastfeeding by delivering not just vital nutrients and probiotics for proper development and bacterial colonization in infants, but also serving as a conduit for commensal microbes and immunological molecules that shape the newborn’s initial microbiota and immune system (Rodríguez et al, 2021). Breastfed infants have higher levels of anti-inflammatory compounds (SCFA, indole-3-lactic acid) and beneficial bifidobacteria species, reducing the incidence of asthma (Davis et al, 2022; Ho et al, 2018; Ciprandi et al, 2023). Probiotic supplementation with Lactobacillus, Streptococcus, and Bifidobacterium is under clinical trial in breastfeeding mothers to evaluate the promotion of beneficial microbiota in infants in reducing necrotizing enterocolitis and late-onset sepsis incidence (NCT06118801).

### Gut-genitourinary tract axis

The genitourinary-gut axis connects the GI tract with organs like the kidneys, bladder, and reproductive organs. This communication involves microbiota, the immune system, hormones, and neural pathways.

#### Urinary tract infections (UTI)

UTI are 20–40 times more prevalent in women than in men of the same age (Deltourbe et al, 2022). Although the role of gut microbiota in UTI susceptibility remains unclear, a recent multi-omics study on women with and without a history of recurrent UTIs revealed significant findings. Women with recurrent UTIs had notably lower microbial richness and fewer butyrate-producing bacteria in their gut compared to controls. Additionally, they exhibited different immune responses to bacterial bladder colonization (Worby et al, 2022). In a randomized clinical trial involving 252 postmenopausal women with recurrent UTI, treatment with oral *L. rhamnosus* GR-1 and *L. reuteri* RC-14, either alone or combined with Trimethoprim as an antibiotic prophylaxis, resulted in a reduced rate of recurrent UTIs with no observed antibiotic resistance (Beerepoot et al, 2012).

#### Polycystic ovary syndrome (PCOS)

PCOS is a condition affecting women with hormonal imbalances and ovarian cysts involving gut-genitourinary interactions. Dysbiosis in the gut can lead to inflammation and immune dysregulation, associated with PCOS. Chronic low-grade inflammation is a hallmark of PCOS and is associated with insulin resistance, a common feature of the syndrome. The gut microbiota influences metabolism and insulin sensitivity via mechanisms like SCFA production (Li et al, 2022a; Liu et al, 2017). Clinical trials explored Lactobacillus (NCT04029805), as well as undefined or defined bacterial consortia, including *B. lactis*, *L. salivarius*, and *L. acidophilus*, with inulin to alleviate PCOS symptoms (NCT03325023, NCT04593459). They can be administered in combination with inulin, promoting the growth of lactic acid bacteria and the consequent secretion of antimicrobial peptides such as bacteriocins (Messaoudi et al, 2013; Roberfroid et al, 2010).

#### Prostate cancer

Certain bacterial species can metabolize estrogen, androgen, or their precursors, thereby affecting systemic hormone levels. For instance, mice on a diet rich in *L. reuteri* exhibited a reduced systemic inflammatory state through decreased IL-17 and increased serum testosterone levels (Poutahidis et al, 2014). In contrast, Clostridium can transform gut glucocorticoids into androgens, which may synergistically contribute to the progression of prostate cancer (Ridlon et al, 2013). In a preclinical study, a combination of patient-derived prostate-specific microbiota with anti-PD-1 immunotherapy increased survival and decreased tumor burden in orthotopic MYC- and PTEN-mutant prostate cancer models (Anker et al, 2018). Clinical trials are currently testing FMT from participants who respond to Pembrolizumab in metastatic castration-resistant prostate cancer (NCT04116775).

### Gut-cardiovascular axis

The gut microbiota connection to cardiovascular tissue is pivotal in cardiovascular diseases (CVD). GI microbiota influences host metabolism and inflammation, crucial to CVD pathophysiology. A key player is trimethylamine N-oxide (TMAO), a bacterial metabolite produced from dietary nutrients such as choline, phosphatidylcholine, and carnitine (Bui et al, 2023). Elevated TMAO levels correlate with increased CVD risk, including atherosclerosis, coronary artery disease, and stroke (Huang et al, 2023).

#### Hypertension

Hypertension is a major risk factor for CVD (Sabbatini and Kararigas, 2020). Gut dysbiosis is marked by reduced bacterial diversity and an altered Firmicutes:Bacteroidetes ratio. This results in decreasing production of SCFA (Yang et al, 2015) and increasing TMAO, promoting inflammation within the blood vessels (Yuan et al, 2023). A current clinical trial is investigating the effects, safety, and underlying mechanisms of FMT via oral capsules on primary hypertension (NCT05608447).

#### Atherosclerosis

Atherosclerosis is a form of arteriosclerosis which involves the gradual narrowing of artery walls due to the accumulation of atheromatous plaques. Ecologic® Barrier, a multispecies probiotic containing Bifidobacterium, Lactobacillus, and Lactococcus species, has shown promising results in preventing atherosclerosis in obese postmenopausal women by reducing TMAO and the chronic inflammatory reactions promoted by obesity, and increasing the total antioxidant status (NCT03100162) (Szulińska et al, 2018).

### Gut-skin axis

Recent dermatological research has expanded beyond topical treatments, recognizing the influence of the gut microbiota on skin conditions like acne, eczema, psoriasis, and rosacea. This understanding of the gut-skin axis offers potential for innovative therapeutic strategies targeting both gut and skin to enhance overall health and alleviate skin disorders.

#### Skin damage

Research exploring the impact of UVB radiation on skin injury revealed that the combination of *L. fermentum* strain TKSN041 with nicotinamide mononucleotide effectively scavenges different oxidant species. Additionally, this combination boosts antioxidant capacity by elevating superoxide dismutase, catalase and IL-10 levels, while reducing TNF-α levels in both serum and skin (Zhou et al, 2021).

#### Atopic dermatitis

For AD treatment, gut administration of *B. adolescentis*, promoted T_reg_ differentiation, suppressed Th2 response and increased Lactobacillus relative abundance, which produces propionic acid that correlates with disease amelioration (Fang et al, 2020). Additionally, the efficacy of FMT in patients with AD (NCT04283968), alopecia (NCT04238091), and psoriatic arthritis (NCT04924270) is being investigated.

#### Skin cancer

Certain gut bacterial species like Bacteroides and Bifidobacterium enhanced the effectiveness of immunotherapies in melanoma cancer models (Sivan et al, 2015; Matson et al, 2018). A consortium of 11 strains representing rare, low-abundance components of the human microbiota has shown to induce IFN-γ-producing CD8 + T cells in a melanoma animal model when combined with an anti-PD1 immune checkpoint inhibitor (Tanoue et al, 2019). Finally, other strategies based on FMT showed an enhancement of the immune function, making individuals more receptive to immunotherapy in melanoma (NCT05502913) (Baruch et al, 2021).

### Metabolic diseases

In some cases, metabolic diseases are caused by the accumulation of metabolites. Genetically modified organisms administered on the GI tract with specifically upregulated enzymatic activities can be used as treatment.

#### Phenylketonuria

This condition is characterized by elevated phenylalanine levels. *E. coli* Nissle strains SYNB1618 and SYNB1934 were engineered to express phenylalanine ammonia-lyase, L-amino acid deaminase, and a phenylalanine transporter, facilitating phenylalanine degradation in the GI tract (Isabella et al, 2018). This approach was safe and increased Phe-derived metabolites in gut and urine samples (NCT03516487, NCT04534842). Further advancements involve the directed evolution of phenylalanine ammonia-lyase to enhance efficacy (Adolfsen et al, 2021). Additionally, auxotrophy was induced by deleting a gene involved in cell wall construction, demonstrating safety in trials (NCT04984525).

#### Hiperhomocystinuria

This genetic disorder is characterized by cystathionine beta-synthase dysfunction and methionine accumulation, leading to liver fibrosis and systemic symptoms. *E. coli* Nissle SYB135 has been engineered to express methionine importers and, through the deletion of exporters, to retain methionine, in combination with enzymes capable of methionine decarboxylation (Perreault et al, 2024). Clinical trials demonstrated the safety and efficacy of SYNB1353 in reducing methionine levels in healthy patients (NCT05462132) (Perreault et al, 2024).

#### Hyperoxaluria

Another metabolic disease treated similarly is enteric hyperoxaluria, caused by excessive accumulation of oxalate leading to renal lithiasis. *E. coli* Nissle SYNB8802 was developed by introducing synthetic circuits capable of degrading oxalate into formate (NCT04629170) (Lubkowicz et al, 2022).

## Conclusions

Genome engineering technologies have advanced rapidly, enabling simultaneous modifications across multiple genomic loci in diverse species, marking a paradigm shift in genetic manipulation. Molecular biology techniques, particularly CRISPR-Cas systems, offer precise and efficient genome editing capabilities. This progress has facilitated the exploration of new species for biomedical applications and the modification of previously inaccessible bacteria.

Current approaches in LBP utilize both pathogenic and commensal bacteria, providing novel alternatives either for diseases lacking effective treatments or for non-responsive patients. Engineered LBP can be tailored for specific therapeutic goals, offering advantages such as well-understood mechanisms of action and delivery strategies.

Leveraging the gut microbiota to modulate immune responses and metabolic processes shows promise for developing new treatment strategies for various disorders.

Emerging trends in LBP research include advanced delivery systems to enhance therapy efficacy and specificity, integration into combined therapies with other modalities, and personalized approaches based on patient-specific microbiota profiles. Ensuring the safety of LBP is paramount, requiring meticulous preclinical evaluation, safety testing and strategies for enhancing containment and control.

In conclusion, while engineered LBP holds significant promise for treating human diseases, addressing safety concerns and navigating regulatory challenges are essential for achieving their full potential in clinical practice. Continued research and rigorous clinical trials will play a vital role in shaping the future of this innovative therapeutic approach.

## Supplementary information

Dataset EV1

Dataset EV2

Dataset EV3
